# Dr. Huggan, on Tapping at the Navel

**Published:** 1807-03

**Authors:** A. Huggan


### *To the Editor of the Medical and Phyſical Journal.*

GENTLEMEN,

AT a late consultation, in company with Sir Christopher Pegge and my friend Mr. Hemsted, on the case of a female patient with ascites, who, it was agreed, should be tapped, our conversation turned upon the subject of *tapping at the navel*: but without much argument, either for or against it, we all seemed to think that tapping at the usual place, from the ease with which it could be done, was still unobjectionable, and of course did not intend to deviate from it in the present instance. However, when Mr. Hemsted and I met for the purpose, the navel being so prominent, he proposed performing it there, by way of experiment. As it succeeded so much to my expectation at least, and as the advantages of it appeared to me so obvious, I cannot help taking the liberty, with your permission, of briefly submitting them to the consideration of such of your surgical readers as may not, for the reasons assigned above, or for any other, be disposed to adopt it.

In the first place, it can be done with greater ease to the operator, and much less pain to the patient, a *lancet* *only*

*only* being necessary for the purpose, and simply the *skin* to be cut through.

2dly, The flow of the water is promoted by the action of the abdominal muscles, without the necessity of keeping an instrument in the wounded part for that purpose.

3dly, When the abdomen is tapped at the usual place, the flow of the water is often interrupted by the omentum, or some other part, plugging up the instrument retained in the opening. From the situation of the Navel, this inconvenience (sometimes very distressing to the patient) is not likely to happen. Besides, the expert surgeon will always be able to adapt his pressure, so as to form a prominence at the navel, which may obviate that, and at the same time keep up the flow as long as it may be necessary. The water flows faster (if that is any advantage) by an opening at the navel, than at the usual place.

It may also be added here, by way of argument, that in tapping at the usual place, the epigastric artery has been wounded, (instances of which are certainly recorded by authors) a circumstance which must, whenever it does happen, be very embarrassing to the Surgeon, and probably fatal to the patient. This accident, though it has happened but seldom, ought nevertheless to be taken into the calculation of occurrences as not improbable. This inconvenience, if that was all, may indeed be as certainly obviated by tapping in the linea alba as at the navel. Though I never saw this operation performed there, I cannot but think it must be attended with nearly the same inconveniencies as when it is done at the usual place; the pain to the patient will be the same; the same instrument, viz. the trocar, must be used, and the canula of it left in the wound to keep it open, while the water is flowing; the same interruption will as frequently take place; nor will the Surgeon be able, by any pressure he can make, to form a prominence, as at the navel, to prevent this interruption, or keep up the stream till it is emptied. It may be said indeed, that if the linea alba is tapped half way between the umbilicus and pubes, where it is recommended to be done, the opening being more dependant, will be more favourable to the flow of the water than at the navel. This, however, in practice, from the posture in which the patient is commonly placed, is a circumstance hardly worth the attending to; at least, in the case above mentioned, the water appeared to me to have been as completely emptied, as it could have been by an opening made in any other part of the abdomen.

